# Migraine susceptibility is modulated by food triggers and analgesic overuse via sulfotransferase inhibition

**DOI:** 10.1186/s10194-022-01405-z

**Published:** 2022-03-14

**Authors:** Doga Vuralli, Burak Arslan, Elif Topa, Andreia Lopes de Morais, Ozlem Gulbahar, Cenk Ayata, Hayrunnisa Bolay

**Affiliations:** 1 Department of Neurology and Algology, Neuropsychiatry Center, Neuroscience and Neurotechnology Center (NÖROM), Gazi University Faculty of Medicine, Besevler, Ankara, Turkey; 2 Neurovascular Research Lab, Department of Radiology, Massachusetts General Hospital, Harvard Medical School, MA Charlestown, USA; 3 Department of Medical Biochemistry, Gazi University Faculty of Medicine, Besevler, Ankara, Turkey; 4 Neuropsychiatry Center, Gazi University, Besevler, Ankara, Turkey; 5 Stroke Service and Neuroscience Intensive Care Unit, Department of Neurology, Massachusetts General Hospital, Harvard Medical School, MA Charlestown, USA

**Keywords:** SULT1A1, Migraine food triggers, Medication overuse, Cortical spreading depression

## Abstract

**Background/aim:**

Certain constituents in migraine food triggers and non-steroidal anti-inflammatory drugs (NSAIDs) inhibit sulfotransferases (SULTs) that detoxify drugs/chemicals and play role in the metabolism of neurotransmitters. We aimed to dissect SULT1A1 modulation of CSD susceptibility and behavior in an in vivo experimental model using hesperidin, a SULT1A1 inhibitor found in citrus fruits (known migraine triggers) and mefenamic acid (SULT1A1 inhibitor), an NSAID to simulate medication overuse.

**Methods:**

Hesperidin was used as SULT1A1 inhibitor found in citrus fruits, known migraine triggers and mefenamic acid (NSAID), another SULT1A1 inhibitor, was used to induce MO in rats. The groups were; 1) Hesperidin (ip) or its vehicle-DMSO (ip) 2) Chronic (4 weeks) mefenamic acid (ip) or its vehicle (ip) 3) Chronic mefenamic acid+hesperidin (ip) or DMSO (ip). CSD susceptibility was evaluated and behavioral testing was performed. SULT1A1 enzyme activity was measured in brain samples.

**Results:**

Single-dose of hesperidin neither changed CSD susceptibility nor resulted in any behavioral change. Chronic mefenamic acid exposure resulted in increased CSD susceptibility, mechanical-thermal hypersensitivity, increased head shake, grooming and freezing and decreased locomotion. Single dose hesperidin administration after chronic mefenamic acid exposure resulted in increased CSD susceptibility and mechanical-thermal hypersensitivity, increased freezing and decreased locomotion. SULT1A1 enzyme activity was lower in mefenamic acid and mefenamic acid+hesperidin groups compared to their vehicles.

**Conclusion:**

Mefenamic acid and hesperidin have synergistic effect in modulating CSD susceptibility and pain behavior. Sulfotransferase inhibition may be the common mechanism by which food triggers and NSAIDs modulate migraine susceptibility. Further investigations regarding human provocation studies using hesperidin in migraine patients with medication overuse are needed.

## Introduction

Migraine is a neurovascular disorder with recurrent headache attacks and signs and symptoms of hypersensitivity to mutiple sensory stimuli accompanying these attacks. It is triggered by various factors in genetically susceptible individuals. Headache attacks are accompanied by nausea and vomiting and hypersensitivity to visual, auditory, olfactory and tactile stimuli. Medication overuse usually underlies the chronification of migraine and medication overuse headache affects approximately 1–2% of the population [1].

It is known that stress, noise, hunger, insomnia and various foods such as chocolate, citrus, coffee, tea and red wine trigger migraine. Although migraine food triggers are well defined, it is still not shown how they start a migraine attack. Some substances in these migraine triggers inhibit a group of enzymes known as sulfotransferases. Sulfotransferases are involved in detoxification of drugs, chemicals and metabolism of neurotransmitters. For example, quercetin and catechin in chocolate, hesperidin in orange and lemon, quinic and caffeic acids in coffee, epicatechin gallate in tea and red wine cause inhibition in SULT1A1 and SULT1A3 enzymes [2]. SULT1A enzymes are also involved in the metabolism of hormones and neurotransmitters such as catecholamines especially dopamine. Several previous studies have shown decreased sulfotransferase activities in platelets in migraine patients [3–6]. Littlewood et al. reported that sulfotransferase activity in platelets was lower in migraine patients who had a history of migraine attacks with migraine food triggers compared to non-dietary migraine and healthy controls [7]. Migraine triggers could initiate a migraine attack by inhibiting SULT1A enzymes, which have a role in deactivation of neurotransmitters, thus, inhibition of these enzymes would cause the levels of neurotransmitters such as cathecolamines including dopamine to fluctuate and may alter migraine susceptibility.

Non-steroidal anti-inflammatory drugs (NSAIDs) used in acute migraine attacks such as mefenamic acid, diflunisal, nimesulide, diclofenac, salicylic acid, ketoprofen, indomethacin, ibuprofen, ketorolac and naproxen have also been shown to inhibit SULT1A1 enzymes [8]. Medication overuse usually occurs in migraine patients. Medication overuse by inhibiting these enzymes could make the migraine patients more susceptible to triggers and triggers even in subthreshold amounts could result in chronic daily headache.

Cortical hyperexcitability has been shown in animal models of medication overuse headache (MOH). When cortical spreading depression (CSD) is induced with topical KCl 1 hour after acute paracetamol (200 mg/kg, intraperitoneal) administration, c-fos activation was reduced in the parietal cortex and trigeminal nucleus caudalis (TNC) but CSD frequency was not altered whereas, chronic paracetamol administration (200 mg/kg daily dose for 30 days, intraperitoneal) caused an increase in c-fos activation and CSD frequency [9]. CSD threshold was also shown to be reduced in another animal model of MOH where sumatriptan was administered by a subcutaneous pump for 6 days [10]. In another experimental study using functional magnetic resonance imaging in rats, after 7 days of sumatriptan administration with osmotic minipumps, bright light stress produced CSD-like responses in sumatriptan group but not in the control group [11]. Medication overuse may increase the sensitivity of migraine patients to triggers, worsen the headache attacks and increase the headache frequency and since both NSAIDs and migraine food triggers inhibit SULT1A enzymes, SULT1A inhibition may be one of the underlying mechanisms for the modulation of migraine susceptibility by such food items and NSAIDs.

We hypothesized that SULT1A inhibition was the common mechanism by which food triggers and NSAIDs modulate migraine susceptibility, in a way that to explain food triggers of migraine attacks and medication overuse (MO) headache. Since both NSAIDs and food triggers inhibit SULT1A enzymes, in migraine patients with medication overuse, SULT1A involvement would cause increased susceptibility to triggers by changing cortical excitability where subthreshold triggers would be enough to cause headache. SULT1A3 is only found in primates and humans but not in rodents and other lower order animals [12, 13]. Therefore, we aimed to dissect SULT1A1 modulation of CSD susceptibility and behavior in an in vivo experimental model using hesperidin, a SULT1A1 inhibitor found in citrus fruits (known migraine triggers) and mefenamic acid (SULT1A1 inhibitor), an NSAID to simulate medication overuse.

## Materials and methods

### Animals

Male Sprague Dawley rats weighing 285–507 g were used in all experiments and were housed in a climate controlled environment and a dark/light cycle of 12 h of daylight and 12 h of darkness with ad libitum access to food and water. Electrophysiological and behavioral studies were performed in separate groups of rats. All procedures were conducted in compliance with the National Institutes of Health (NIH) guidelines for use of laboratory animals and institutional guidelines for animal care and use for research purposes were strictly followed and the study was approved by institutional review board.

### Experimental groups

Hesperidin 100 mg/kg was used as SULT1A1 inhibitor found in citrus fruits, known migraine triggers and mefenamic acid (NSAID) 20 mg/kg, another SULT1A1 inhibitor, was used to induce medication overuse. In chronically treated rats, body weight was monitored weekly throughout the treatment period. Animals were randomly assigned to experimental groups.

### Cortical spreading depression studies

CSD susceptibility was assessed by measuring the electrical threshold for CSD and the CSD frequency during continuous topical application of KCl in the experimental groups below. Other CSD attributes such as propagation speed, amplitude and duration at half-maximal amplitude were also measured.

1. Hesperidin 100 mg/kg (*n* = 16) or its vehicle-DMSO (*n* = 16) was administered intraperitoneally (ip) 30 min prior to CSD experiments.
2. Mefenamic acid 20 mg/kg/day (*n* = 12) or its vehicle-5% DMSO and sesame oil (*n* = 12) was administered (ip) chronically for 4 weeks and on day 28, CSD susceptibility was examined.
3. Mefenamic acid 20 mg/kg/day was administered (ip) for 4 weeks and on day 28, 30 min prior to CSD experiments, single dose hesperidin 100 mg/kg (*n* = 12) or its vehicle-DMSO (*n* = 12) was given intraperitoneally.

### Behavioral studies

Behavioral tests were performed in a different set of experimental groups with intact skull.

1. Hesperidin 100 mg/kg (*n* = 12) or its vehicle-DMSO (*n* = 12) was administered intraperitoneally (ip) 30 min prior to behavioral studies.
2. Mefenamic acid 20 mg/kg/day (*n* = 12) or its vehicle-5% DMSO and sesame oil (*n* = 12) was administered (ip) chronically for 4 weeks and on day 28, behavioral tests were performed.
3. Mefenamic acid 20 mg/kg/day was administered (ip) for 4 weeks and on day 28, hesperidin 100 mg/kg (*n* = 12) or its vehicle-DMSO (*n* = 12) was given (ip) 30 min prior to behavioral studies.

### General surgical preparation

Rats were anesthetized with isoflurane, the femoral artery was cannulated and a tracheostomy tube was placed for mechanical ventilation (SAR-830; CWE, Ardmore, PA). Rectal core temperature was maintained at 37 °C with a heating pad. Continuous blood pressure monitoring was established via femoral artery catheter (ADInstruments, Ardmore, PA) and intermittent arterial pH, pCO2 and O2 were measured (Rapidlab 248 blood gas/pH analyzer; Siemens HealthCare, Eschborn, Germany) and maintained within physiological range by adjusting ventilation parameter settings.

### Cortical spreading depression susceptibility testing 

Animals were placed on a stereotactic frame (Stoelting, Wood Dale, IL), craniotomies were drilled over the occipital (mm from bregma: 4.5 posterior, 2 lateral; diameter 2 mm), parietal (mm from bregma 1.5 posterior, 2 lateral,1 mm diameter), and frontal cortices (mm from bregma 1.5 anterior, 2 lateral; diameter 1 mm) under saline irrigation and dura was removed. Glass capillary microelectrodes (approximately 250 mm deep) were inserted and the cortex was allowed to rest for 15 min under saline. The electrocorticogram and direct current potentials were recorded and signals were amplified (EX1 differential amplifiers; Dagan Corporation, Minneapolis, MN) and recorded continuously (PowerLab; ADInstruments, Colorado Springs, CO).

**Fig. 1:**
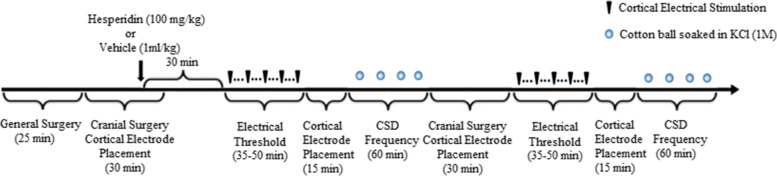
Experimental timeline to test the effect of hesperidin or its vehicle on electrical CSD threshold and CSD frequency. All symbols are defined on the right upper corner

Susceptibility to CSD was assessed by measuring the electrical threshold for CSD and the frequency of CSD during continuous topical KCl application [14, 15]. The electrical threshold for CSD was determined by direct stimulation of the cortex with a bipolar stimulation electrode (400 mm tip diameter, 1 mm tip separation; Frederick Haer Company, Bowdoin, ME) placed on the cortical pial surface and a stimulus isolator (WPI, Sarasota, FL). Single-square pulses of increasing duration and intensity (50–4000 mC) were applied at 5-min intervals until a CSD was triggered at the recording site. CSD frequency was analysed using a cotton ball (1.5–2 mm diameter) soaked with KCl (1 M) placed on the occipital cortex and the cotton ball was changed every 15 min and CSDs were continuously recorded for an hour. CSDs with an amplitude ≥5 mV were counted. Propagation speed, amplitude, and duration (at halfmaximal amplitude) of CSDs were also measured. Electrical thresholds and KCl induced CSD frequencies were measured in both hemispheres. Experimental set up is shown on Fig. 1.

### Behavior

Behavioral tests were conducted at the same time period of the day. Spontaneous behavior such as grooming, freezing, immobility and head shakes was recorded for 10 min in plexiglass cages where rats were free to move. Elevated plus maze was used to assess anxiety like responses [16, 17]. The rats were placed at the center of the elevated plus maze set up facing the same closed arm and their spontaneous behavior was recorded for 5 min with the vide-camera sistem on the setup. Each animal was tested only once. The maze was cleaned with 70% alcohol after each animal. Duration spent in open and closed times and the number of open and closed arm entries are recorded.

Periorbital mechanical withdrawal thresholds were evaluated with von Frey filaments using ‘up and down method’ [18] starting with a force of 4 g. First, rats were allowed to accommodate to 40 cm high platforms and then von Frey filaments were applied to the periorbital region to assess mechanical withdrawal thresholds. Avoidance of rats from von Frey filament or ipsilateral head grooming was considered as a positive response.

Acetone evaporation test was performed to assess cold allodynia. One drop of acetone was applied to the periorbital region and the test was repeated 5 times with 5-min intervals. Grooming and avoidance within 1 min is considered as a positive response. Positive response was expressed as a percentage.

### SULT 1A1 enzyme activity detection

At the end of the behavioral testing (2 h after the administration of hesperidin or its vehicle ip), the animals were sacrified by thiopental (50 mg/kg). Brains were harvested and rat brains were stored at − 80 °C. A spectrophotometric assay was used to study SULT1A1 enzyme activity where p-nitrophenyl sulfate was the sulfate donor and PAP was the sulfate acceptor molecule forming PAPS and p-nitrophenyl anion, which could be measured at 405 nm.

### Homogenization

On the study day, the samples were taken from − 80 °C freezer and weighed one by one on a precision scale and the weights of the tissues were noted. For homogenization, tissue samples were diluted with homogenization buffer (%1.15 KCl, 1 mM EDTA ve 1 μg/mL protease inhibitor (aprotinin) containing 10 mM HEPES-NaOH, pH 7.4) in a ratio of 1/5. Brain tissues were homogenized in a mortar on ice. After homogenization, it was centrifuged at > 9000×g to obtain post-mitochondrial fraction in a refrigerated centrifuge. The supernatant obtained after centrifugation was placed in appropriate eppendorf tubes and stored at − 80 °C until the time of the analysis. On the analysis day, the eppendorf tubes were dissolved at + 4 °C.

### Reagents

Assay buffer: 50 mM NaCl containing 25 MOPS buffer, pH 7.2, and 1 mM EDTA.

PAP reagent: 2 mM stock solution (8.6 mg/10 mL) in the assay buffer was prepared.

p-Nitrophenyl sulfate reagent: 2 mM (5.2 mg/10 mL) in the assay buffer was prepared.

#### Analysis and calculation

**Fig. 2:**
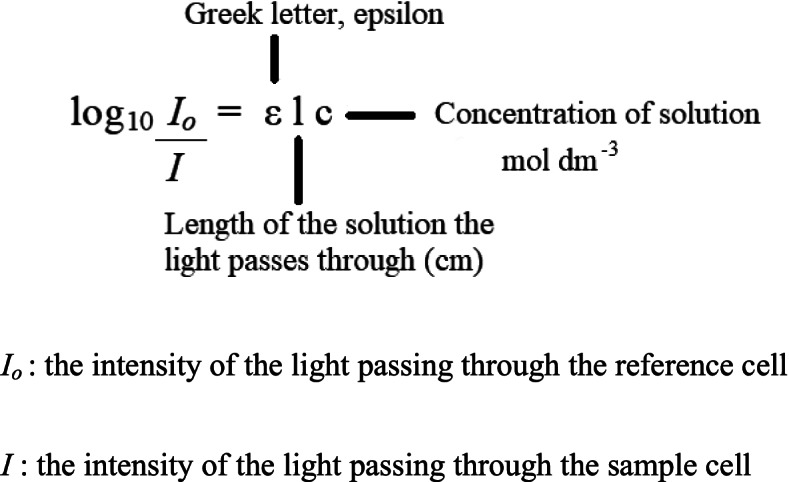
The absorbance of the solution with epsilon value was calculated using the equation above according to Beer Lambert law

In a clean microplate or borosilicate tube, 50 μL of S10 (10,000×g supernatant for 20 min at 4 °C) or water, 25 μL of PAP reagent and 25 μL of p-nitrophenyl sulphate were pipetted and they were completed to 250 μL with assay buffer (150 μL). Reagents and samples were placed in 96 well plate. The formation of p-nitrophenyl anion was measured over 30 min at 405 nm at 10 min intervals with a plate reader. All absorbance values measured at 10 min intervals from t: 0 to t: 30 were recorded. According to Beer Lambert law, the activity of the solution with epsilon value was calculated according to the equation in Fig. 2. Epsilon value was taken as 15,000 M^− 1^ cm^− 1^ at 405 nm [19]. Obtained initial value was converted to IU (international unit = μmol/min) and other calculations were done based on it [20]. The specific enzyme activity was calculated with respect to the protein concentration (mg/mL).

The length of the solution in the equation was taken as the length of the well of the plate which was 0.5 cm. Calculation of enzyme activity was based on average absorbance values. Delta average absorbances and enzyme activities were calculated according to the equations below;

$$\mathrm{Delta}\ \mathrm{average}\ \mathrm{absorbance}=\left(\mathrm{A}\left(\mathrm{t}10-\mathrm{t}0\right)+\mathrm{A}\left(\mathrm{t}20-\mathrm{t}10\right)+\mathrm{A}\left(\mathrm{t}30-\mathrm{t}20\right)\right)/3\ \mathrm{where}\ \mathrm{A}\ \mathrm{is}\ \mathrm{absorbance}\ \mathrm{and}\ \mathrm{t}\ \mathrm{is}\ \mathrm{time}.$$

$$\mathrm{Enzyme}\ \mathrm{Activity}\left(\mathrm{IU}/\mathrm{L}\right)=\mathrm{Delta}\ \mathrm{Average}\ \mathrm{Absorbance}\ \left(10-0,20-10,30-20\ \min \right)\ \mathrm\times\ 2.1\times1{0}^4$$

### Study design and statistics

All experiments were performed and analyzed in a blinded fashion. A priori exclusion criteria were surgical failure and poor systemic physiology. None of the animals died or experienced severe complications during the CSD recordings. Data were presented as mean ± standard deviation or as bar graphs. Analyses were made by Prism 7 (GraphPad Software, Inc., CA, USA). Continuous variables were analyzed with Student t-tests, or 2-way analysis of variance for repeated measures and post-hoc multiple comparisons were carried out using Šidák’s multiple comparisons test. A *P* value of 0.05 was considered statistically significant.

## Results

Systemic physiological parameters were within normal limits in all anesthetized rats and were not different between the groups (Table 1).

**Table 1:** Systemic physiology

Experimental Groups	Body weight (g)	BP (mmHg)	pH	pCO_**2**_ (mmHg)	pO_**2**_ (mmHg)
Single dose DMSO	378.6 ± 53.0	98.0 ± 7.1	7.42 ± 0.03	34.0 ± 2.0	151.6 ± 16.0
Single dose hesperidin	365.4 ± 62.0	99.0 ± 10.0	7.42 ± 0.03	33.7 ± 2.5	152.2 ± 13.8
Chronic 5%DMSO+sesame oil	459.0 ± 27.4	98.2 ± 8.3	7.45 ± 0.03	34.5 ± 2.7	154.6 ± 11.0
Chronic mefenamic acid	446.8 ± 24.8	100.3 ± 14.7	7.43 ± 0.02	34.4 ± 2.3	163.2 ± 7.8
Chronic mefenamic acid + single dose DMSO	434.2 ± 30.3	97.0 ± 7.7	7.42 ± 0.03	34.1 ± 2.2	147.0 ± 7.7
Chronic mefenamic acid + single dose hesperidin	424.3 ± 33.5	90.0 ± 7.9	7.43 ± 0.04	33.4 ± 1.8	156.8 ± 9.5

### Effect of hesperidin, mefenamic acid and mefenamic acid+hesperidin on cortical spreading depression susceptibility

**Fig. 3:**
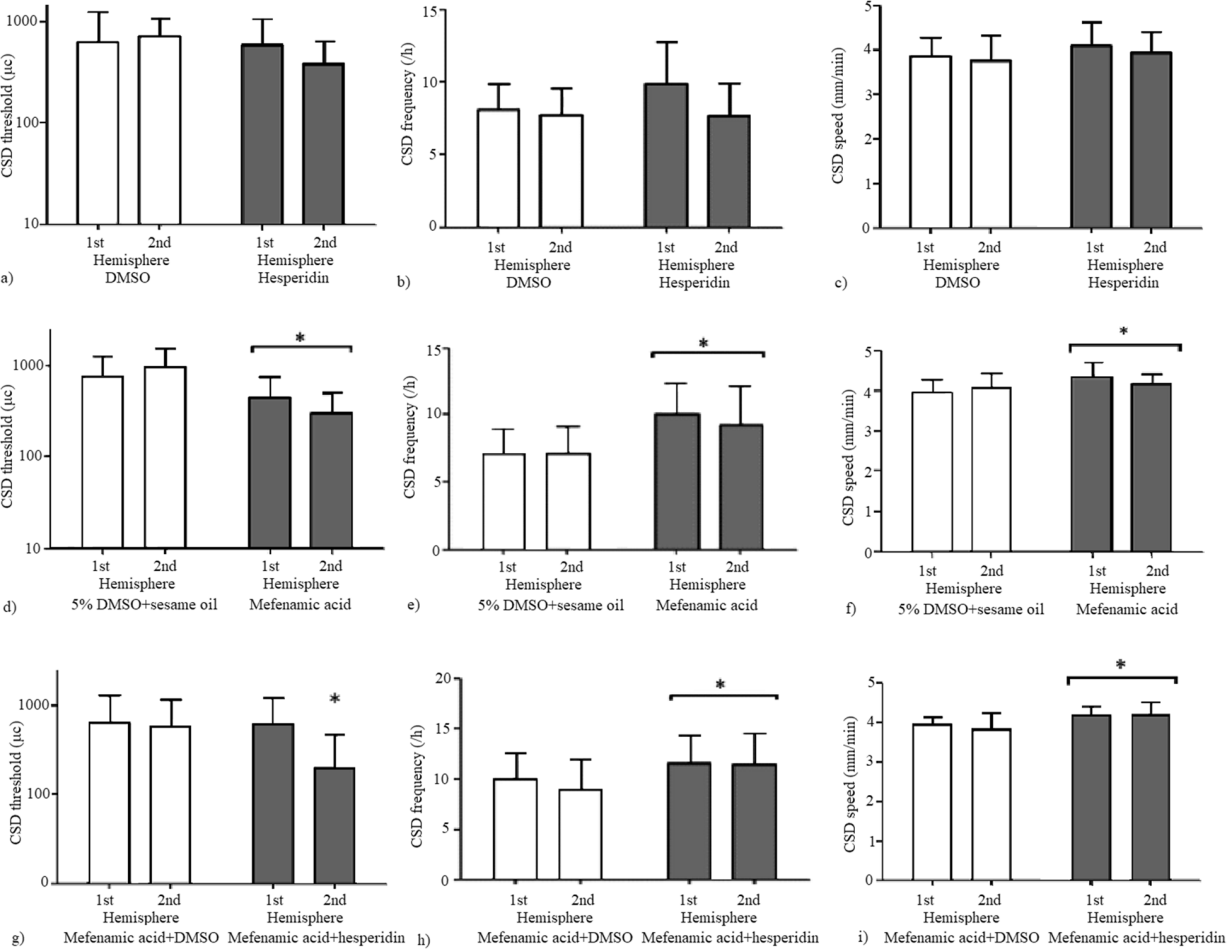
Hesperidin 100 mg/kg (ip) 30 min before testing did not alter **a**) the cortical electrical stimulation thresholds, **b**) the frequency of CSDs triggered by 1 h continuous topical KCl application or **c**) CSD speed compared to its vehicle DMSO (*p* = 0.21, *p* = 0.22 and *p* = 0.16 respectively). Four week chronic mefenamic acid administration resulted in decreased **d**) electrical thresholds for CSD (*p* = 0.0033) and **e**) increased KCl induced CSD frequency and **f**) CSD speed (*p* = 0.0059 and *p* = 0.0218 respectively) compared to its vehicle 5% DMSO+sesame oil. A single dose of hesperidin further increased CSD susceptibility in chronically mefenamic acid treated rats compared to its vehicle DMSO. **g**) The electrical thresholds for CSD were significantly lower in the second hemisphere (*p* = 0.048) and **h**) CSD frequencies and **i**) CSD speed were significantly higher (*p* = 0.04 and *p* = 0.0012 respectively) in both hemispheres in chronically mefenamic acid treated rats that received a single dose of hesperidin on day 28 compared to its vehicle DMSO. Vertical axis of CSD threshold is in log scale. *p < 0.05 vs. controls

**Fig. 4:**
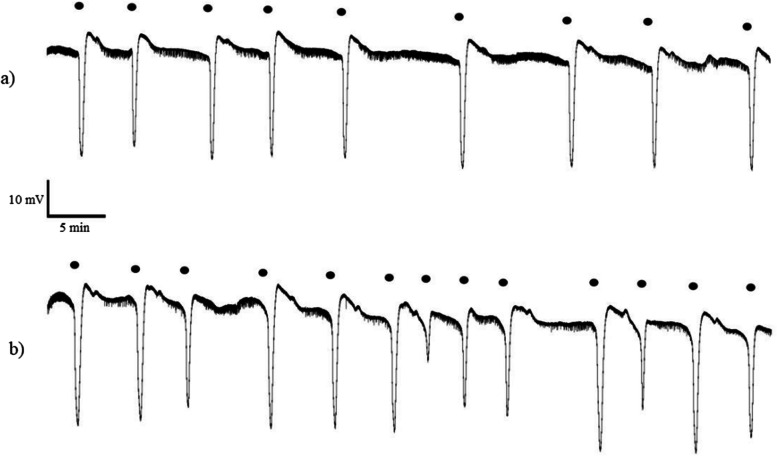
Representative intracortical microelectrode recordings provided from chronic mefenamic acid admistrated rats followed by **a**) a single dose of DMSO or **b**) a single dose of hesperidin on day 28

Hesperidin 100 mg/kg (ip) 30 min before testing did not alter CSD attributes compared to its vehicle DMSO. The cortical electrical stimulation thresholds, the frequency of repetitive CSDs triggered by 1 h continuous topical KCl application and propagation speed of CSD were similar between hesperidin and DMSO groups (*p* = 0.21, *p* = 0.22 and *p* = 0.16 respectively). Four week chronic mefenamic acid administration increased CSD susceptibility on day 28 compared to its vehicle 5% DMSO+sesame oil (Fig. 3). The electrical thresholds for CSD were significantly lower (*p* = 0.0033) and KCl induced CSD frequencies/hour and propagation speed of CSD were significantly higher (*p* = 0.0059 and *p* = 0.0218 respectively) in both hemispheres in rats receiving chronic mefenamic acid compared to its vehicle (Fig. 3). Four week chronic mefenamic acid administration and a single dose of hesperidin on day 28 resulted in increased CSD susceptibility compared to chronic mefenamic acid plus single dose DMSO group (Fig. 3). The electrical thresholds for CSD were significantly lower in the 2. hemisphere (*p* = 0.048) and KCl induced CSD frequencies and CSD speed were significantly higher (*p* = 0.04 and *p* = 0.0012 respectively) in both hemispheres in rats receiving chronic mefenamic acid plus a single dose of hesperidin on day 28 compared to chronic mefenamic acid plus DMSO group (Fig. 4). Hesperidin and mefenamic acid compared to their vehicles and chronic mefenamic acid plus a single dose of hesperidin compared to chronic mefenamic acid plus a single dose of DMSO had no effect on CSD amplitude (*p* = 0.18, *p* = 0.8 and *p* = 0.5 respectively) or duration  (*p* = 0.4, *p *= 0.4 and *p* = 0.17 respectively) (Table 2).

**Table 2:** Amplitude and duration of the first CSD detected at the posterior electrode

Experimental group	Hemisphere	Duration (sec)	Amplitude (mV)
DMSO	1st	29.4 ± 7.1	21.3 ± 7.8
	2nd	22.8 ± 4.2	22.7 ± 4.9
Hesperidin	1st	27.1 ± 10.4	24.3 ± 3.2
	2nd	22.5 ± 5.0	18.5 ± 7.7
5% DMSO+sesame oil	1st	25.0 ± 6.4	24.3 ± 4.4
	2nd	22.9 ± 4.4	21.5 ± 6.5
Mefenamic acid	1st	24.7 ± 3.1	25.8 ± 3.8
	2nd	25.4 ± 5.3	20.6 ± 7.3
Mefenamic acid+DMSO	1st	24.0 ± 5.8	24.1 ± 4.7
	2nd	25.3 ± 5.0	22.3 ± 7.0
Mefenamic acid+hesperidin	1st	27.3 ± 8.2	25.1 ± 2.1
	2nd	28.1 ± 6.0	23.7 ± 3.9

### Effect of hesperidin, mefenamic acid and mefenamic acid+hesperidin on behavior

**Fig. 5:**
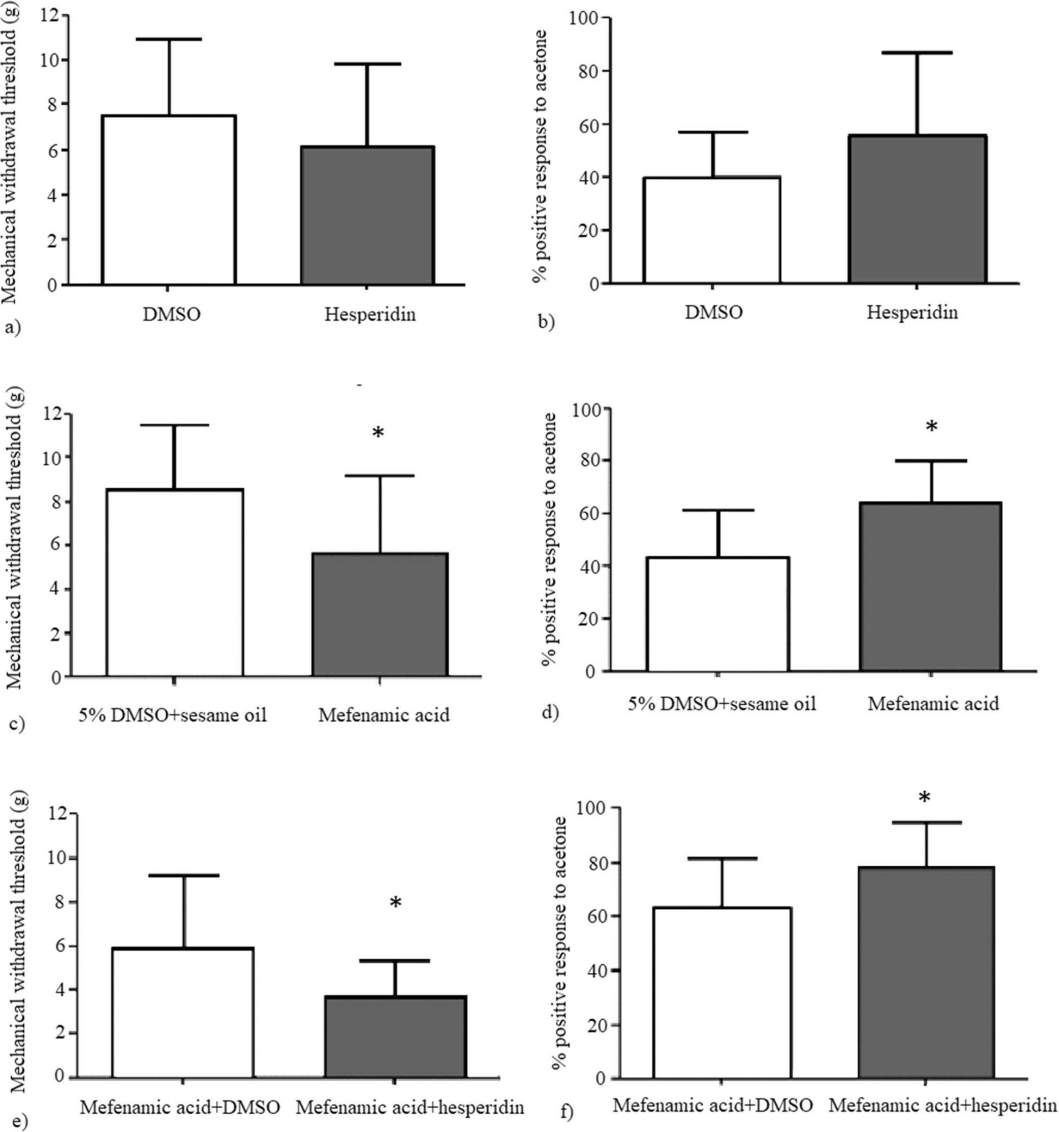
No statistically significant difference was found between hesperidin and DMSO groups regarding **a**) the mechanical withdrawal thresholds (*p* = 0.34) and **b**) the percent positive response to acetone (*p* = 0.34). **c**) The mechanical withdrawal thresholds were lower (*p* = 0.038) and **d**) the percent positive responses to acetone were higher (*p* = 0.007) in chronic mefenamic acid group compared to its vehicle (5% DMSO+sesame oil). **e**) The mechanical withdrawal thresholds were lower (*p* = 0.047) and **f**) the percent positive responses to acetone were higher (*p* = 0.046) in chronic mefenamic acid plus hesperidin group compared to chronic mefenamic acid plus DMSO group. *p < 0.05 vs. controls

**Fig. 6:**
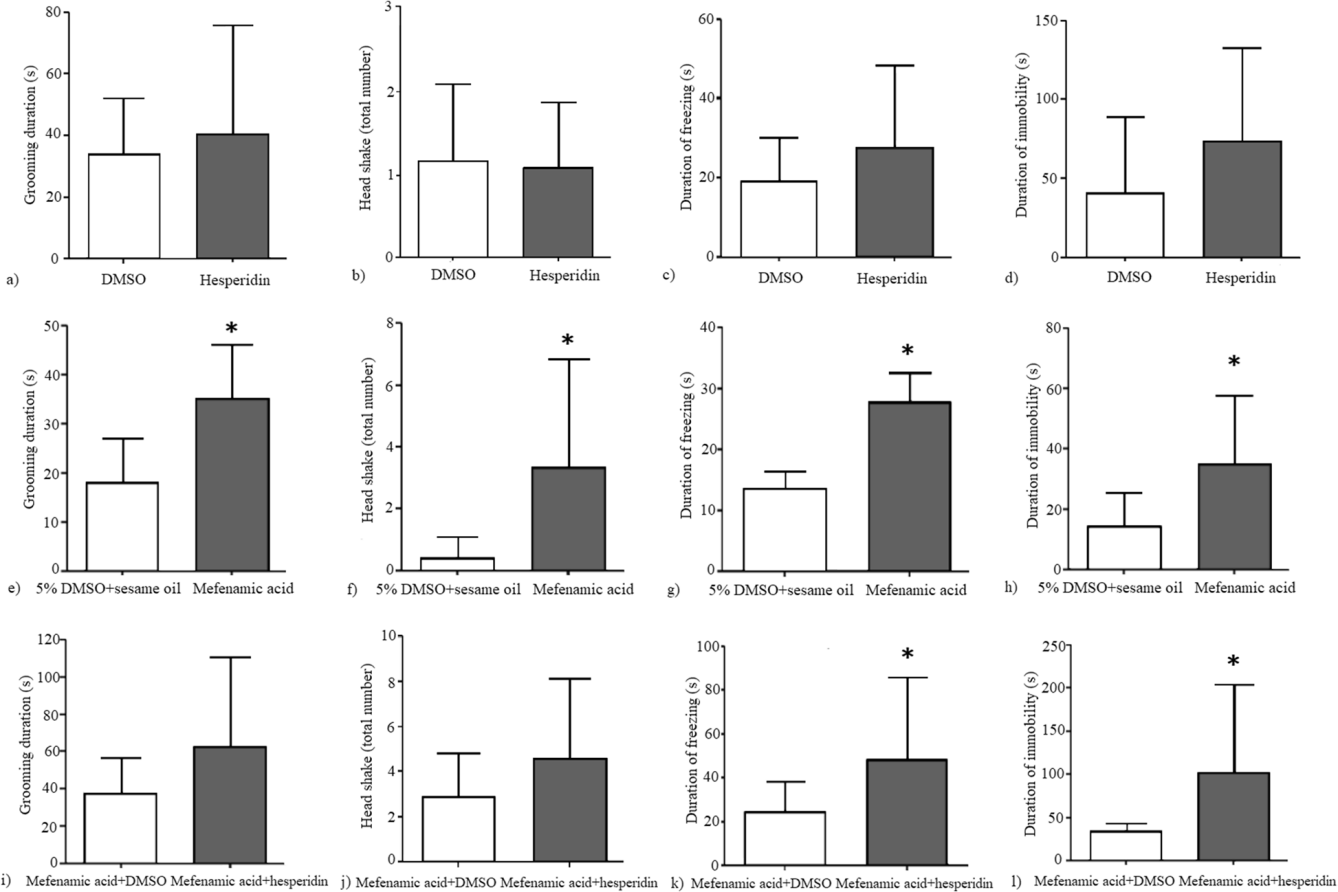
No statistically significant difference was found between hesperidin and DMSO groups regarding the **a**) duration of grooming (*p* = 0.6) **b**) the number of head shakes (*p* = 0.82). **c**) duration of freezing (*p* = 0.22) and **d**) duration of immobility (*p* = 0.146). **e**) Grooming duration, **f**) number of head shakes **g**) duration of freezing, and **h**) duration of immobility were higher in the chronic mefenamic acid group compared to its vehicle (*p* = 0.001, *p* = 0.01, *p* = 0.018 and *p* = 0.01 respectively). **i**) Grooming duration and **j**) number of head shakes were similar between the chronic mefenamic acid plus hesperidin and chronic mefenamic acid plus DMSO groups (*p* = 0.113, *p* = 0.17, respectively) while **k**) duration of freezing (*p* = 0.048) and **l**) duration of immobility (*p* = 0.03) were higher in chronic mefenamic acid plus hesperidin group. *p < 0.05 vs. controls

**Fig. 7:**
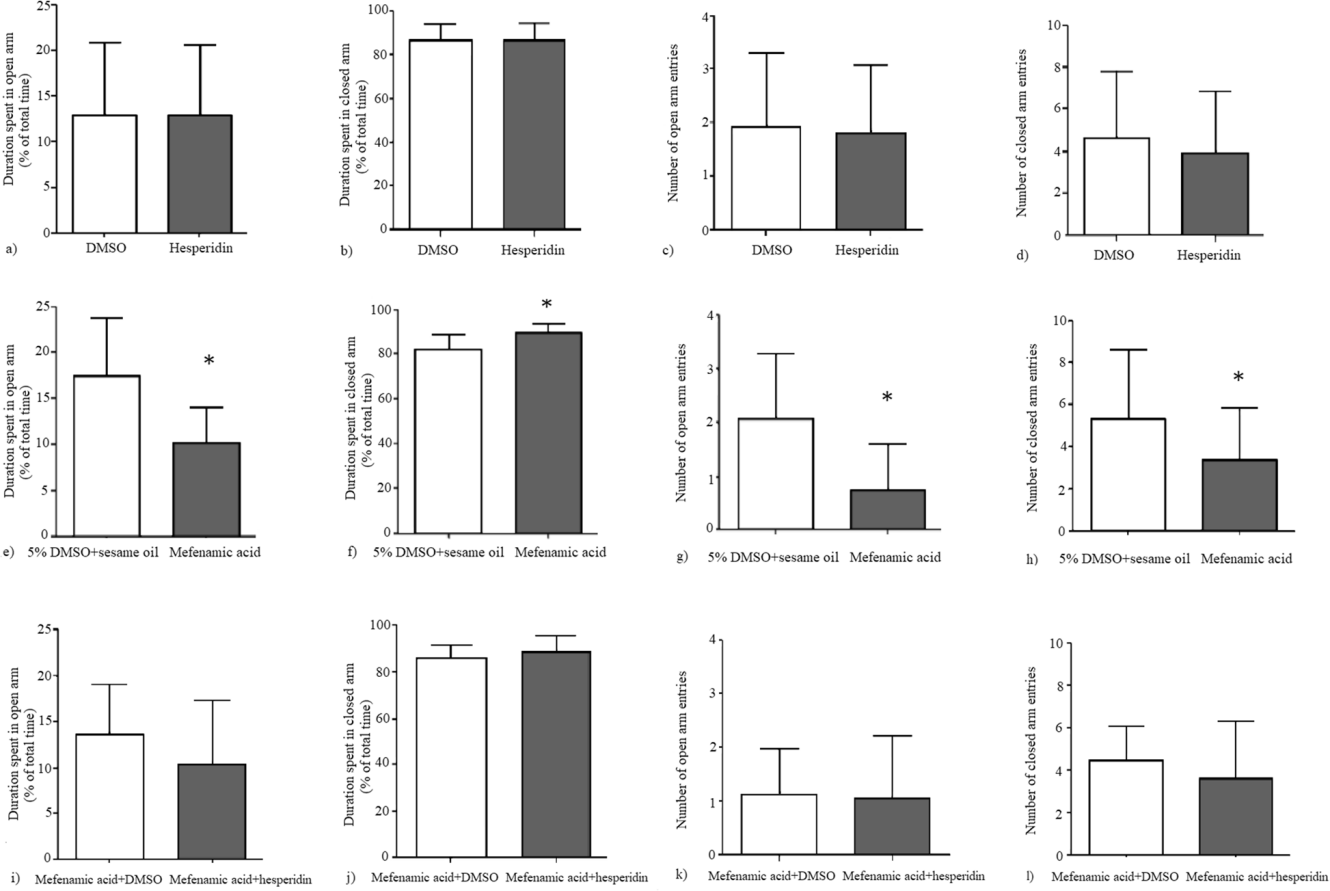
In elevated plus maze test, **a**) duration spent in open and **b**) closed arms (*p* = 0.99 and *p* = 0.99, respectively) and **c**) the number of open and **d**) closed arm entries were similar between hesperidin and DMSO groups (*p* = 0.88 and *p* = 0.6, respectively). **e**) Duration spent in open arms was lower (*p* = 0.003) and **f**) duration spent in closed arms was higher (*p* = 0.003) and **g**) number of open arm entries were lower (*p* = 0.006) and **h**) number of closed arm entries were similar (*p* = 0.12) in the elevated plus maze test in the chronic mefenamic acid group compared to its vehicle. **i**) Duration spent in open and **j)** closed arms and **k**) the number of open and **l**) closed arm entries were comparable between chronic mefenamic acid plus hesperidin and chronic mefenamic acid plus DMSO groups (*p* = 0.23, *p* = 0.32, *p* = 0.84, *p* = 0.37 respectively). *p < 0.05 vs. controls

Hesperidin alone did not result in any significant behavioral change. There was no statistically significant difference between hesperidin and DMSO groups according to the durations of grooming (*p* = 0.6), freezing (*p* = 0.22) and immobility (*p* = 0.146), mechanical withdrawal thresholds (*p* = 0.34) and the percent positive response to acetone (*p* = 0.34) (Fig. 5). In elevated plus maze test, duration spent in open and closed arms (*p* = 0.99 and *p* = 0.99, respectively) and the number of open and closed arm entries were not statistically different between hesperidin and DMSO groups (*p* = 0.88 and *p* = 0.6, respectively). The mechanical withdrawal thresholds were significantly lower (*p* = 0.038) and the percent positive responses to acetone were significantly higher (*p* = 0.007) in chronic mefenamic acid group compared to its vehicle (5% DMSO+sesame oil) (Fig. 5). Grooming duration, number of head shakes and duration of freezing and duration of immobility were significantly higher in the chronic mefenamic acid group compared to its vehicle (*p* = 0.001, *p* = 0.01, *p* = 0.018, *p* = 0.01, respectively) (Fig. 6). Duration spent in open arms was significantly lower (*p* = 0.003) and duration spent in closed arms was significantly higher (*p* = 0.003) and number of open arm entries were significantly lower (*p* = 0.006) in the elevated plus maze test in the chronic mefenamic acid group compared to its vehicle (Fig. 7). Number of closed arm entries were similar (*p* = 0.12) between chronic mefenamic acid and 5%DMSO+sesame oil groups (Fig. 7). The mechanical withdrawal thresholds were significantly lower (*p* = 0.047) and the percent positive responses to acetone were significantly higher (*p* = 0.046) in chronic mefenamic acid plus hesperidin group compared to chronic mefenamic acid plus DMSO group (Fig. 5). Duration of freezing (*p* = 0.048) and duration of immobility (*p* = 0.03) were significantly higher in chronic mefenamic acid plus hesperidin group while grooming duration and number of head shakes were similar (Fig. 6) between the two groups (*p* = 0.113, *p* = 0.17, respectively). In elevated plus maze test, duration spent in open and closed arms and the number of open and closed arm entries were similar (Fig. 7) between chronic mefenamic acid plus hesperidin and chronic mefenamic acid plus DMSO groups (*p* = 0.23, *p* = 0.32, *p* = 0.84, *p* = 0.37 respectively).

#### Brain SULT1A1 enzyme activity levels

**Fig. 8:**
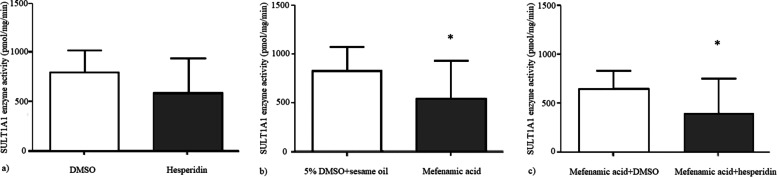
**a**) Brain SULT1A1 enzyme activities were comparable between DMSO and hesperidin groups (*p* = 0.1) while **b**) SULT1A1 enzyme activity was lower in chronic mefenamic acid group compared to its vehicle (*p* = 0.044) and **c**) it was also lower in chronic mefenamic acid plus hesperidin group compared to chronic mefenamic acid plus DMSO group (*p* = 0.048). *p < 0.05 vs. controls

Brain SULT1A1 enzyme activities were not significantly different between DMSO and hesperidin groups (*p* = 0.1) while SULT1A1 enzyme activity was significantly lower in chronic mefenamic acid group compared to its vehicle (*p* = 0.044) and it was also significantly lower in chronic mefenamic acid plus hesperidin group (Fig. 8) compared to chronic mefenamic acid plus DMSO group (*p* = 0.048).

## Discussion

We showed for the first time that mefenamic acid and hesperidin had a synergistic effect in modulating CSD susceptibility and pain behavior via SULT1A1 inhibition. Four week exposure to mefenamic acid resulted in increased CSD susceptibility, mechanical and thermal hypersensitivity, increased head shake, grooming and freezing time and decreased locomotion revealing central sensitization and pain-like behavior consistent with medication-overuse headache. Single dose hesperidin administration after 4-week exposure to mefenamic acid resulted in a synergistic effect on CSD modulation and pain behavior and caused further increase in CSD susceptibility and mechanical and thermal hypersensitivity and decreased locomotion and increased freezing while hesperidin alone neither changed CSD susceptibility nor resulted in any behavioral change.

Enhanced CSD susceptibility and cortical hyperexcitability may explain the mechanism by which medication overuse causes clinical worsening of headache frequency and severity in migraine patients with medication overuse headache. Even though chronic administration of acute migraine abortive treatments such as triptans and acetaminophen has been shown to enhance cortical excitability, affect CSD threshold and frequency and facilitate the trigeminal nociceptive process in animal studies, there is no preclinical study on the impact of chronic NSAID exposure on CSD susceptibility. After 30-day administration of acetaminophen, increased CSD frequency and CSD-evoked expression of Fos in trigeminal nucleus caudalis were shown in rats, indicating a facilitation of trigeminal nociception [9]. In another study, 6 day exposure to sumatriptan markedly reduced the electrical threshold required to elicit CSD and bright stress induced behavioral withdrawal thresholds [10]. Furthermore, 6 day sumatriptan exposure increased CSD-induced Fos expression in the TNC consistent with heightened activation of the trigeminal pathway [10]. Similarly, we showed that chronic mefenamic acid exposure decreased the electrical stimulation threshold to generate CSD and increased KCl induced CSD frequency. Chronic mefenamic acid exposure resulted in pain-like behaviors such as increased head shake, grooming, freezing and decreased locomotion. Moreover, chronic mefenamic acid administration led to decreased mechanical withdrawal thresholds and increased sensitivity to acetone in the periorbital region consistent with facilitation of the trigeminal nociceptive process shown in previous studies of medication overuse models. Freezing behavior has been shown to be a behavioral marker for head pain in animal models of migraine [21–24]. Increased grooming is also associated with increased discomfort and nociception [22, 25, 26]. Head shakes have been shown to be significantly increased by CSDs [22]. Mechanical allodynia has been reported in different studies using nitroglycerin-induced model of migraine [27, 28]. The acetone evaporation test assesses cold allodynia and increased acetone sensitivities in the forehead and facial region were shown in animal models of chronic migraine [29, 30]. An increase in sensitivity of trigeminal afferents (peripheral sensitization) and central trigeminal neurons (central sensitization) have pivotal roles in chronic migraine and medication overuse headache and involve inflammatory mediators. In medication overuse animal models, upregulation of vasoactive and inflammatory mediators such as calcitonin gene–related peptide (CGRP), substance P, and nitric oxide synthase have been shown in the trigeminal ganglia [31, 32]. The peripheral sensitization underlies cutaneous allodynia observed in migraine and animal studies revealed a similar effect of chronic analgesic use on trigeminal afferents. Repeated or continuous administration of triptans resulted in tactile allodynia as shown by decreased periorbital and hind paw thresholds in rats and increased the number of CGRP positive neurons in the trigeminal ganglia [33]. Similarly, in our study, chronic mefenamic acid administration resulted in reduced periorbital mechanical withdrawal thresholds consistent with mechanical allodynia and increased acetone sensitivity showing cold allodynia.

Analgesic overuse is known to deteriorate headache in patients with migraine. Cortical hyperexcitability, central and peripheral sensitization caused by medication overuse as shown in animal studies may lead to increased sensitivity to triggers. Acute migraine attacks are often treated with NSAIDs and excessive use of these medications may lead to medication overuse headache that may cause increased sensitivity and responsiveness to triggers resulting in increased frequency of migraine attacks. Since both NSAIDs and food triggers inhibit SULT1A enzymes, in migraine patients with medication overuse, SULT1A involvement may cause increased susceptibility to triggers by changing cortical excitability where subthreshold triggers would be enough to cause headache. Hesperidin, a SULT inhibitor found in citrus fruits, which are known migraine triggers, alone neither changed CSD susceptibility nor resulted in any behavioral change. However, hesperidin after chronic mefenamic acid exposure further decreased electrical threshold for CSD and increased the frequency and speed of CSD. Additionally hesperidin enhanced pain-like behavior in chronic mefenamic acid treated rats. Hesperidin further decreased the periorbital mechanical withdrawal thresholds and increased acetone sensitivity in rats that received chronic mefenamic acid suggesting a more pronounced increase in cortical and trigeminal excitability. Mefenamic acid and hesperidin have a synergistic effect in modulating CSD susceptibility and pain behavior. SULT1A1 inhibition may be the common mechanism by which food triggers and NSAIDs modulate CSD and migraine susceptibility.

Anxiety-like behavior has been shown in headache animal models. In a chronic migraine animal model, the percentage of open arm entries was significantly lower in chronic migraine group compared to controls which supported increased anxiety-like behavior [34]. Similarly, in our study, in rats receiving chronic mefenamic acid, the decrease in the number of open arm entries, the shortening of the duration spent in open arm and the prolongation of duration spent in the closed arm were observed indicating increased anxiety-like behavior. In a previous study, chronic exposure to analgesics was found to increase the excitability of neurons in amygdala [35], which may underly the anxiety-like behavior we observed in chronic mefenamic acid group.

Migraine triggers and NSAIDs may modulate migraine susceptibility by inhibiting sulfotransferase enzymes. Migraine is known to be provoked by some food items such as chocolate, citrus fruits, wine and cheese. Flavonoids are found in these migraine food triggers and they inhibit SULT1A enzymes. Flavonoids function as pro-oxidants when they are in high concentrations or oxidated by intracellular enzymes such as myeloperoxidase or when they come in contact with iron and copper. Repeated migraine attacks were shown to be related to iron deposition in putamen, globus pallidus, red nucleus and periaqueductal gray matter. Nitrogylcerin, a migraine trigger, was shown to impair cellular iron trafficking and increase CGRP receptors in meningeal cells [36]. In the presence of free iron in brain not sequestered in ferritin or by microglia, flavonoids may act as pro-oxidants locally [37]. SULT1A1 values were shown to be lower in dietary migraine subjects compared to non-dietary migraine group and healthy controls [7, 38]. Reduced SULT1A1 enzyme activity was shown in migraineurs compared to tension-type headache patients and healthy control subjects [3, 5]. The inhibition of sulfotransferase enzymes by phenolic flavonoids could also result in buildup of free phenols in the circulation, which may be toxic in several ways and less detoxification of migraine-precipitating substances in diet and fluctuations in neurotransmitters such as cathecolamines, especially dopamine.

SULTs in the gastrointestinal tract and in the brain eliminate cathecolamines by sulfonation and prevents excess entry of catecholamines to systemic circulation and modulates their brain levels. Inhibition of these enzymes would alter the levels of these neurotransmitters and may facilitate migraine headache attacks. Dopamine is thought to play a role in migraine headache [39]. Dopamine receptor hypersensitivity has been shown in patients with migraine [40–43]. Intense yawning, craving and altered sensory perception during the initial phase of the migraine attack are some of the clinical evidence of dopamine involvement in migraine attacks [43]. D2 receptor blockade prevents the prodromal symptoms [39]. The efficacy of D2 antagonists in the acute treatment of migraine is supported by both clinical and preclinical studies [44–46]. Platelet [47] and plasma levels of dopamine [48, 49] and dopamine metabolite levels in cerebrospinal fluid were higher in migraine patients compared to healthy controls [47] suggesting an abnormal metabolism of dopamine in migraine [50]. Unbalanced levels of dopamine and noradrenaline in the pain matrix may be associated with the activation of the trigeminal system leading to a migraine attack [51]. The relationship between dopamine levels and SULT1A1 inhibition, CSD and migraine susceptibility remains to be established.

We showed for the first time that low SULT1A1 enzyme activity is associated with enhanced CSD susceptibility and pain like behavior in rats. In a medication overuse rat model with chronic mefenamic acid exposure, we found lower brain SULT1A1 enzyme activity levels. Moreover, this is the first study to investigate the impact of chronic NSAID exposure on CSD susceptibility and pain behavior. A common clinical problem in migraine patients with medication overuse is the clinical deterioration with increased frequency and severity of headache and acute exacerbations probably induced even with subtreshold triggers. SULT1A1 inhibition could be one of the underlying mechanisms of this clinical deterioration that is seen commonly in migraine patients with medication overuse. Further increase in CSD susceptibility with hesperidin after chronic mefenamic acid exposure may explain the increased sensitivity and responsiveness to triggers resulting in increased frequency of migraine attacks in patients with medication overuse. These results should be confirmed in humans by provoking migraine attacks with hesperidin in migraine patients with medication overuse.

## Conclusion

Chronic analgesic use lowers the threshold for CSD and renders the brain more susceptible to the effects of migraine triggers. Triggers that cannot initiate a migraine attack alone may be able to initiate an attack when the brain is more susceptible due to chronic analgesic use. Hesperidin further reduced CSD thresholds, increased CSD frequencies, decreased mechanical withdrawal thresholds and increased percent positive responses to acetone and increased immobility and freezing time when administered to chronically mefenamic acid treated rats. SULT1A1 inhibition as a common mechanism by which medication overuse and migraine triggers modulate migraine susceptibility, possibly can help us gain insight into mechanisms relevant to migraine pathophysiology. Therefore, they may provide a new potential target and direction in the treatment.

## Data Availability

The datasets during and/or analysed during the current study available from the corresponding author on reasonable request.

## References

[1] Westergaard ML, Hansen EH, Glümer C, Olesen J, Jensen RH (2014). Definitions of medication-overuse headache in population-based studies and their implications on prevalence estimates: a systematic review. Cephalalgia..

[2] Eagle K (2012). Toxicological effects of red wine, orange juice, and other dietary SULT1A inhibitors via excess catecholamines. Food Chem Toxicol.

[3] Davis BA, Dawson B, Boulton AA, Yu PH, Durden DA (1987). Investigation of some biological trait markers in migraine: deuterated tyramine challenge test, monoamine oxidase, phenolsulfotransferase and plasma and urinary biogenic amine and acid metabolite levels. Headache..

[4] Marazziti D, Palego L, Dell'Osso L, Batistini A, Cassano GB, Akiskal HS (1996). Platelet sulfotransferase in different psychiatric disorders. Psychiatry Res.

[5] Alam Z, Coombes N, Waring RH, Williams AC, Steventon GB (1997). Platelet sulphotransferase activity, plasma sulphate levels and sulphation capacity in patients with migraine and tension headache. Cephalalgia..

[6] Jones AL, Roberts RC, Colvin DW, Rubin GL, Coughtrie MW (1995). Reduced platelet phenolsulphotransferase activity towards dopamine and 5-hydroxytryptamine in migraine. Eur J Clin Pharmacol.

[7] Littlewood J, Glover V, Sandler M, Petty R, Peatfield R, Rose FC (1982). Platelet phenolsulphotransferase deficiency in dietary migraine. Lancet..

[8] Vietri M, De Santi C, Pietrabissa A, Mosca F, Pacifici GM (2000). Inhibition of human liver phenol sulfotransferase by nonsteroidal anti-inflammatory drugs. Eur J Clin Pharmacol.

[9] Supornsilpchai W, le Grand SM, Srikiatkhachorn A (2010). Cortical hyperexcitability and mechanism of medication-overuse headache. Cephalalgia..

[10] Green AL, Gu P, De Felice M, Dodick D, Ossipov MH, Porreca F (2014). Increased susceptibility to cortical spreading depression in an animal model of medication-overuse headache. Cephalalgia..

[11] Becerra L, Bishop J, Barmettler G, Xie Y, Navratilova E, Porreca F (2016). Triptans disrupt brain networks and promote stress-induced CSD-like responses in cortical and subcortical areas. J Neurophysiol.

[12] Sidharthan NP, Minchin RF, Butcher NJ (2013). Cytosolic sulfotransferase 1A3 is induced by dopamine and protects neuronal cells from dopamine toxicity: role of D1 receptor-N-methyl-D-aspartate receptor coupling. J Biol Chem.

[13] Gamage N, Barnett A, Hempel N, Duggleby RG, Windmill KF, Martin JL (2006). Human sulfotransferases and their role in chemical metabolism. Toxicol Sci.

[14] Ayata C (2013). Pearls and pitfalls in experimental models of spreading depression. Cephalalgia..

[15] Ayata C, Jin H, Kudo C, Dalkara T, Moskowitz MA (2006). Suppression of cortical spreading depression in migraine prophylaxis. Ann Neurol.

[16] Pellow S, File SE (1986). Anxiolytic and anxiogenic drug effects on exploratory activity in an elevated plus-maze: a novel test of anxiety in the rat. Pharmacol Biochem Behav.

[17] Sayin A, Derinöz O, Yüksel N, Şahin S, Bolay H (2014). The effects of the estrus cycle and citalopram on anxiety-like behaviors and c-fos expression in rats. Pharmacol Biochem Behav.

[18] Chaplan SR, Bach FW, Pogrel JW, Chung JM, Yaksh TL (1994). Quantitative assessment of tactile allodynia in the rat paw. J Neurosci Methods.

[19] Cook I, Wang T, Falany CN, Leyh TS (2013). High accuracy in silico sulfotransferase models. J Biol Chem.

[20] Swinehart DF (1962). The beer-lambert law. J Chem Educ.

[21] Tepe N, Filiz A, Dilekoz E, Akcali D, Sara Y, Charles A (2015). The thalamic reticular nucleus is activated by cortical spreading depression in freely moving rats: prevention by acute valproate administration. Eur J Neurosci.

[22] Filiz A, Tepe N, Eftekhari S, Boran HE, Dilekoz E, Edvinsson L (2019). CGRP receptor antagonist MK-8825 attenuates cortical spreading depression induced pain behavior. Cephalalgia..

[23] Vuralli D, Wattiez AS, Russo AF, Bolay H (2019). Behavioral and cognitive animal models in headache research. J Headache Pain.

[24] Akcali D, Sayin A, Sara Y, Bolay H (2010). Does single cortical spreading depression elicit pain behaviour in freely moving rats?. Cephalalgia..

[25] Stucky NL, Gregory E, Winter MK, He YY, Hamilton ES, McCarson KE (2011). Sex differences in behavior and expression of CGRP-related genes in a rodent model of chronic migraine. Headache..

[26] Melo-Carrillo A, Lopez-Avila A (2013). A chronic animal model of migraine, induced by repeated meningeal nociception, characterized by a behavioral and pharmacological approach. Cephalalgia..

[27] Bates EA, Nikai T, Brennan KC, Fu YH, Charles AC, Basbaum AI (2010). Sumatriptan alleviates nitroglycerin-induced mechanical and thermal allodynia in mice. Cephalalgia..

[28] Farkas S, Bölcskei K, Markovics A, Varga A, Kis-Varga Á, Kormos V (2016). Utility of different outcome measures for the nitroglycerin model of migraine in mice. J Pharmacol Toxicol Methods.

[29] Akçali D, Belen AD, Babacan A, Bolay H (2017). Nitroglycerin challenge induces lateralized headache in nasociliarynerve-ligated rats: implications for chronic migraine. Turk J Med Sci.

[30] Kim SJ, Yeo JH, Yoon SY, Kwon SG, Lee JH, Beitz AJ (2018). Differential development of facial and hind paw Allodynia in a nitroglycerin-induced mouse model of chronic migraine: role of capsaicin sensitive primary afferents. Biol Pharm Bull.

[31] Belanger S, Ma W, Chabot JG, Quirion R (2002). Expression of calcitonin gene-related peptide, substance P and protein kinase C in cultured dorsal root ganglion neurons following chronic exposure to mu, delta and kappa opiates. Neuroscience..

[32] De Felice M, Ossipov MH, Wang R, Dussor G, Lai J, Meng ID (2010). Triptan-induced enhancement of neuronal nitric oxide synthase in trigeminal ganglion dural afferents underlies increased responsiveness to potential migraine triggers. Brain..

[33] De Felice M, Ossipov MH, Wang R, Lai J, Chichorro J, Meng I (2010). Triptan-induced latent sensitization: a possible basis for medication overuse headache. Ann Neurol.

[34] Zhang M, Liu Y, Zhao M, Tang W, Wang X, Dong Z (2017). Depression and anxiety behaviour in a rat model of chronic migraine. J Headache Pain.

[35] Wanasuntronwong A, Jansri U, Srikiatkhachorn A (2017). Neural hyperactivity in the amygdala induced by chronic treatment of rats with analgesics may elucidate the mechanisms underlying psychiatric comorbidities associated with medication-overuse headache. BMC Neurosci.

[36] Aral LA, ErgÜn MA, Bolay H (2021). Cellular iron storage and trafficking are affected by GTN stimulation in primary glial and meningeal cell culture. Turk J Biol.

[37] Borkum JM (2016). Migraine triggers and oxidative stress: a narrative review and synthesis. Headache..

[38] Launay JM, Soliman H, Pradalier A, Dry J, Dreux C (1988). Platelet phenol sulfotransferase activities: a migraine marker?. Therapie..

[39] Charbit AR, Akerman S, Goadsby PJ (2010). Dopamine: what's new in migraine?. Curr Opin Neurol.

[40] Gorji A (2001). Spreading depression: a review of the clinical relevance. Brain Res Brain Res Rev.

[41] Del Zompo M, Lai M, Loi V, Pisano MR (1995). Dopamine hypersensitivity in migraine: role in apomorphine syncope. Headache..

[42] Blin O, Azulay JP, Masson G, Aubrespy G, Serratrice G (1991). Apomorphine-induced yawning in migraine patients: enhanced responsiveness. Clin Neuropharmacol.

[43] Blau JN (1992). Migraine: theories of pathogenesis. Lancet..

[44] Peroutka SJ (1997). Dopamine and migraine. Neurology..

[45] Marmura MJ (2012). Use of dopamine antagonists in treatment of migraine. Curr Treat Options Neurol.

[46] Doğanay Aydin H, Vuralli D, Akçali DT, Bolay H (2017). Metoclopramide inhibits trigeminovascular activation:evidence for effective acute attack treatment in migraine. Turk J Med Sci.

[47] D'Andrea G, Granella F, Perini F, Farruggio A, Leone M, Bussone G (2006). Platelet levels of dopamine are increased in migraine and cluster headache. Headache..

[48] D'Andrea G, D'Arrigo A, Dalle Carbonare M, Leon A (2012). Pathogenesis of migraine: role of neuromodulators. Headache..

[49] Nagel-Leiby S, Welch KM, D'Andrea G, Grunfeld S, Brown E (1990). Event-related slow potentials and associated catecholamine function in migraine. Cephalalgia..

[50] Castillo J, Martínez F, Suárez C, Naveiro J, Lema M, Noya M (1996). Cerebrospinal fluid tyrosine and 3,4-dihydroxyphenylacetic acid levels in migraine patients. Cephalalgia..

[51] D'Andrea G, Leon A (2010). Pathogenesis of migraine: from neurotransmitters to neuromodulators and beyond. Neurol Sci.

